# Evaluation of healthcare-associated infection rates in patients with hematologic malignancies and stem cell transplantation during the coronavirus disease 2019 (COVID-19) pandemic

**DOI:** 10.1017/ash.2021.237

**Published:** 2022-01-17

**Authors:** Laura J. Bobbitt, Gowri Satyanarayana, Laura Van Metre Baum, Caroline A. Nebhan, Adetola A. Kassim, Katie S. Gatwood

**Affiliations:** 1 Department of Pharmaceutical Services, Vanderbilt University Medical Center, Nashville, Tennessee; 2 Division of Infectious Diseases, Department of Medicine, Vanderbilt University Medical Center, Nashville, Tennessee; 3 Division of Medical Oncology, Department of Medicine, Yale University School of Medicine, New Haven, Connecticut; 4 Division of Hematology and Oncology, Department of Medicine, Vanderbilt University Medical Center, Nashville, Tennessee

## Abstract

**Objective:**

To evaluate whether rates of healthcare-associated infections (HAIs) changed during the coronavirus disease 2019 (COVID-19) pandemic in malignant hematology and stem cell transplant patients.

**Design:**

A retrospective, cohort study.

**Patients:**

The study included malignant hematology and stem cell transplant patients admitted between March 1, 2019, through July 31, 2019, and March 1, 2020, through July 31, 2020.

**Methods:**

Rates of catheter-associated urinary tract infections (CAUTIs), central-line–associated bloodstream infections (CLABSIs), central-line–associated mucosal barrier injury infections (CLAMBIs), and *Clostridioides difficile* infections (CDIs) during the pandemic were compared to those in a control cohort. Secondary outcomes included the rate of non–COVID-19 respiratory viruses.

**Results:**

The rate of CAUTIs per 1,000 hospital days was 0.435 before the pandemic and 0.532 during the pandemic (incidence rate ratio [IRR], 1.224; 95% confidence interval [CI], 0.0314–47.72; *P* = .899). The rate of CLABSIs was 0.435 before the pandemic and 1.064 during the pandemic (IRR, 2.447; 95% CI, 0.186–72.18; *P* = .516). The rate of CLAMBIs was 2.61 before the pandemic and 1.064 during the pandemic (IRR 0.408, 95% CI 0.057–1.927; *P* = .284). The rate of CDIs was 2.61 before the pandemic and 1.579 during the pandemic (IRR, 0.612; 95% CI, 0.125–2.457; *P* = .512). Non–COVID-19 respiratory virus cases decreased significantly from 12 (30.8%) to 2 cases (8.3%) (*P* = 0.014).

**Conclusions:**

There was no significant difference in HAIs among inpatient malignant hematology and stem cell transplant patients during the COVID-19 pandemic compared to those of a control cohort. Rates of infection were low among both cohorts. Rates of community-acquired respiratory viruses decreased significantly during the pandemic among this population.

Healthcare-associated infections (HAIs) are infections that patients contract while receiving medical treatment in a healthcare facility. The most common HAIs are catheter-associated urinary tract infections (CAUTIs), central-line–associated bloodstream infections (CLABSIs), and *Clostridioides difficile* infections (CDIs).^1^ Nationally, between 2018 and 2019, there was an ∼8% decrease in CAUTIs in acute-care hospitals, a 7% decrease in CLABSIs, and an 18% decrease in CDIs, reflecting ongoing progress in preventing HAIs.^2^

During the coronavirus disease 2019 (COVID-19) pandemic, there has been an emphasis on infection prevention measures such as isolation and barrier precautions, hand hygiene, and social distancing to prevent the transmission of severe acute respiratory coronavirus virus 2 (SARS-CoV-2). Beginning in May 2020, our institution mandated masks, temperature checks, and symptoms screening on arrival for patients and employees. Significant emphasis was placed on increased handwashing and the use of gloves. Further changes included visitor restrictions to 1 visitor per patient; fewer employees on campus due to remote work options; and more employees wearing scrubs instead of white coats, ties, and any clothing that was not laundered daily. Additionally, due to concerns about shortages of personal protective equipment (PPE), providers minimized entry into patient rooms under contact precautions. Metropolitan Nashville and the State of Tennessee took additional precautions to limit the spread of COVID-19. On April 1, 2020, a stay-at-home order was enacted and Tennesseans were encouraged to stay at home except for essential activities. Restrictions were lifted in a phased approach, with each phase containing specific recommendations related to businesses, gatherings, and travel. Nashville began phase 1 of reopening on May 11, 2020 then moved into phase 2 on May 25, 2020 and into phase 3 on June 22, 2020. On July 3, 2020, the city returned to phase 2 with modifications.^3^

The impact of increased infection control measures on HAIs is unclear, but some have hypothesized that these measures may have the unintended benefit of decreasing HAI rates. A large healthcare system in Singapore reported a significant reduction in CLABSIs during the pandemic compared to a prepandemic cohort but no significant change in the rate of CAUTIs.^4^ Conversely, a retrospective study of CLABSIs at a tertiary-care center in the Greater Detroit area found a statistically significant increase in CLABSIs.^5^ Hospitals in Rome, Italy, and Madrid, Spain, both reported significant decreases in CDI rates during the pandemic.^6,7^

Malignant hematology and stem-cell transplant patient are at an especially high risk for infectious complications due to their immunocompromised state. Studies in stem-cell transplant patients have reported rates of HAIs of 35.5 to 40.6 per 1,000 days of neutropenia.^8–10^ Risk factors for infection within this population include severe and prolonged neutropenia, presence of a central venous catheter, damage to the mucocutaneous barrier caused by mucositis, and the presence of graft-versus-host disease (GVHD).^11^ Standard precautions to prevent HAIs in this patient population, such as hand hygiene and the use of HEPA filtration and positive-pressure rooms, were already in place prior to the COVID-19 pandemic.^12^ Although better prevention and treatment of infectious complications have improved outcomes, infections remain a major cause of morbidity and mortality in this patient population.^8^

No studies to our knowledge have been published on HAI rates in immunocompromised patients during the COVID-19 pandemic. We compared the rates of HAIs in malignant hematology and stem cell transplant patients before the COVID-19 pandemic to rates during the COVID-19 pandemic with the implementation of increased hygiene and infection control practices.

## Methods

### Study design

We conducted a single-center, retrospective, cohort study of malignant hematology and stem cell transplant patients at Vanderbilt University Medical Center (VUMC) in Nashville, Tennessee. The study was approved by the VUMC Institutional Review Board. Adult malignant hematology and stem cell transplant patients admitted between March 1, 2020, through July 31, 2020, and a comparator cohort of patients admitted from March 1, 2019, through July 31, 2019, were included in the study. Patients with COVID-19 infection were excluded from this study. Medical oncology patients were also excluded.

The primary outcomes were the incidences of CAUTIs, CLABSIs, central-line–associated mucosal barrier injury infections (CLAMBIs), and CDIs reported as infections per inpatient hospital days. Infections were defined according to the CDC site-specific infection criterion.^13^ Secondary outcomes included the rate of non–COVID-19 respiratory viral infections, the rate of identifiable cause of neutropenic fever, the proportion of patients who received broad-spectrum antibiotics, antibiotic treatment duration, and infection-related mortality. All data were obtained from the electronic medical record by chart review and data extraction. For the primary outcome of healthcare-associated infection rates, data obtained were compared to institutional infection control data to ensure accuracy.

### Statistical analysis

Statistical analyses were performed using SPSS version 26 software (IBM, Armonk, NY) and OpenEpi version 3.01 software. Categorical variables are reported as a number and percentage. Continuous descriptive variables are reported as a median and interquartile range. Categorical outcomes were compared using χ^2^ and continuous outcomes were compared using the Mann-Whitney *U* test. Rates of infections were compared using incidence rate ratios and a mid-*P* exact test.^4,14^ A *P* value <.05 was considered statistically significant.

## Results

### Study population

Between March 1, 2019, to July 31, 2019, and March 1, 2020, to July 31, 2020, 688 patients were identified for potential inclusion. After further review, 134 medical oncology patients were excluded. The final analysis included 554 patients: 295 were admitted during the prepandemic period and 259 were admitted during the pandemic. Baseline characteristics are described in Table 1. The prepandemic cohort was significantly older than the pandemic cohort: 62 years versus 58 years (*P* < .0005). The study population comprised 49% malignant hematology patients and 51% stem cell transplant patients. The median time from transplant to inpatient admission was 9 days. On admission, 28% of patients were neutropenic and 14% presented with neutropenic fever. More patients in the prepandemic cohort had mucositis than in the pandemic cohort (29% vs 19%; *P* = .004).

**Table 1.** Baseline Characteristics

Baseline Characteristic	Before the Pandemic (n=295)	During the Pandemic (n=259)	*P* Value
Age, y (range)	62 (54–69)	58 (47–66)	<.0005
**Sex**			.432
Male	167 (56.6)	138 (53.3)	
Female	128 (43.4)	121 (46.7)	
**Race**			
White	242 (82.0)	238 (91.9)	.001
Black or African American	34 (11.5)	12 (4.6)	
Asian	11 (3.7)	0 (0)	
Other	4 (1.4)	7 (2.7)	
Declined to answer	4 (1.4)	2 (0.8)	
Hematologic malignancy	148 (50.2)	122 (47.1)	.471
**Stem cell transplant**	147 (49.8)	137 (52.9)	
Allogeneic	60 (20.3)	73 (28.2)	
Autologous	81 (27.4)	59 (22.8)	.171
CAR-T	6 (2.0)	5 (1.9)	
Time from transplant to admission, d (range)	9 (2–153)	9 (0.5–41.5)	.241
Time from last chemotherapy to admission, d (range)	16 (7–28.25)	14 (4–30)	.451
Neutropenic on admission^a^	81 (27.5)	73 (28.2)	.909
Febrile neutropenia on admission	46 (15.6)	31 (12.0)	.219
Acute GVHD on admission	14 (4.7)	7 (2.7)	.209
Mucositis on admission	86 (29.2)	48 (18.5)	.004

Note. CAR-T, chimeric antigen receptor T-cell therapy; GVHD, graft versus host disease.

a ANC < 500 cells/µL.

### Primary and secondary outcomes

Rates of HAIs were not significantly different between the prepandemic and pandemic settings, although rates were low in both cohorts (Table 2). The rate of CAUTIs per 1,000 inpatient hospital days was 0.435 in the prepandemic cohort and 0.532 in the pandemic cohort (incidence rate ratio [IRR], 1.224; 95% confidence interval [CI], 0.0314–47.72; *P* = .899). The rate of CLABSIs was 0.435 in the prepandemic cohort and 1.064 in the pandemic cohort (IRR, 2.447; 95% CI, 0.186–72.18; *P* = .516). Isolated urinary and bloodstream pathogens are listed in Table 3. The rate of CLAMBIs was 2.61 before the pandemic and 1.064 during the pandemic (IRR, 0.408; 95% CI, 0.057–1.927; *P* = .284). The rate of CDIs was 2.61 in the prepandemic cohort and 1.579 in the pandemic cohort (IRR, 0.612; 95% CI, 0.125–2.457; *P* = .512).

**Table 2.** Primary Outcomes

Outcome^a^	Before the Pandemic (n=295)	During the Pandemic (n=259)	Incidence Rate Ratio (95% CI)	*P* Value
CAUTI	0.435	0.532	1.224 (0.0314–47.72)	.899
CLABSI	0.435	1.064	2.447 (0.186–72.18)	.516
CLAMBI	2.61	1.064	0.408 (0.057–1.927)	.284
CDI	2.61	1.579	0.612 (0.125–2.457)	.512

Note. CAUTI, catheter-associated urinary tract infection; CLABSI, central-line–associated bloodstream infection; CLAMBI, central-line–associated mucosal barrier injury infection; CDI, *Clostridioides difficile* infection.

a Infection rates reported in infections per 1,000 inpatient hospital days.

**Table 3.** Urinary and Bloodstream Pathogens

Pathogens Isolated	Before the Pandemic (n=295)	During the Pandemic (n=259)
**CAUTI (n=2)**		
Yeast, not *Candida albicans*	1	0
*Staphylococcus epidermidis*	0	1
**CLABSI (n=3)**		
Bacteroides thetaiotaomicron group	1	0
*Staphylococcus aureus*, methicillin-resistant	0	1
*Viridans streptococcus*, not anginosus group	0	1
**CLAMBI (n=8)**		
*Enterococcus faecalis*	1	0
*Enterococcus gallinarum*	1	0
*Escherichia coli*, ESBL	0	1
*Klebsiella varicola*	1	0
*Viridans streptococcus*, not anginosus group	0	1
Polymicrobial	3	0

Note. CAUTI, catheter-associated urinary tract infection; CLABSI, central-line–associate bloodstream infection; CLAMBI, central-line–associated mucosal barrier injury infection; ESBL, extended-spectrum β-lactamase.

For secondary outcomes, we identified a significant decrease in non–COVID-19 respiratory viral infections, with 12 cases in the prepandemic cohort and 2 cases in the pandemic cohort (*P* = 0.014). Also, 39 patients (13.2%) in the prepandemic cohort had a respiratory viral test, whereas 24 patients (9.3%) in the pandemic cohort were tested. The rates of identifiable cause of neutropenic fever were 17.4% in the prepandemic cohort and 22.6% in the pandemic cohort (*P* = .75). We detected no significant difference in the percentage of patients who received vancomycin, cefepime, piperacillin-tazobactam, or meropenem between the 2 cohorts. The median antibiotic treatment duration was 5 days in the prepandemic cohort and 4 days in the pandemic cohort (*P* = .06). Finally, there was only 1 case of HAI-related mortality, which occurred in the prepandemic cohort (Table 4).

**Table 4.** Secondary Outcomes

Outcome	Before the Pandemic (n=295)	During the Pandemic (n=259)	*P* Value
**Positive respiratory virus panel**	12/39 (30.8)	2/24 (8.3)	.014
Adenovirus	1	0	
Coronavirus 229E	1	0	
Coronavirus NL63	0	1	
Rhinovirus/enterovirus	10	1	
Identifiable cause of neutropenic fever	8/46 (17.4)	7/31 (22.6)	.750
**Received ≥1 dose of**			
Vancomycin	66 (22.3)	50 (16.9)	.376
Cefepime	117 (39.5)	98 (33.2)	.660
Piperacillin-tazobactam	20 (6.8)	17 (6.6)	.919
Meropenem	11 (3.7)	7 (2.4)	.497
Antibiotic treatment duration, d (range)	5.0 (3.0–9.0)	4.0 (3.0–7.5)	.060
Infection-related mortality^a^	0.435	0	.550

a Infection-related mortality reported per 1,000 inpatient hospital days.

## Discussion

In this study, we did not detect a significant difference in CAUTI, CLABSI, CLAMBI, and CDI rates in malignant hematology and stem cell transplant patients during the COVID-19 pandemic compared to a control cohort. However, overall rates of infection were extremely low in both groups, with only 2 CAUTIs and 3 CLABSIs in both cohorts combined.

Wee et al^4^ reported a significant decrease in the rate of CLABSIs during the COVID-19 pandemic, with a rate of 0.83 incidents per 1,000 device days before the pandemic and a rate of 0.20 incidents per 1,000 device days during the pandemic (IRR, 0.24; 95% CI, 0.07–0.57; *P* < .05). Notably, they detected an increase in CLABSI bundle compliance during the pandemic. Despite better compliance with their CAUTI bundle, CAUTI rates remained stable at 1.8 incidents per 1,000 device days. Our study differs from prior studies in that we examined patient populations in which additional infection prevention practices were already in place prior to COVID-19 due to their immunocompromised states. Furthermore, likely due to good adherence to these measures at VUMC, the rate of HAIs was low at baseline within the control cohort.

Ponce-Alonso et al^7^ detected a decrease in the CDI rate from 8.54 per 10,000 patient days during the control period to 2.68 per 10,000 patient days during the COVID-19 pandemic (*P* = .000257). Bentivegna et al^6^ reported that the CDI incidence was significantly lower compared to 2017 (odds ratio [OR], 2.98; *P* = .002), 2018 (OR, 2.27; *P* = .023) and 2019 (OR, 2.07; *P* = .047). Our study was likely underpowered to show a significant difference in this outcome given the overall low rate of CDIs in our cohort.

To our knowledge, no study has reported rates of CLAMBIs during the COVID-19 pandemic. We did not find a significant difference in rates of CLAMBIs, though we were likely underpowered due to low rates of CLAMBIs. Furthermore, more acute GVHD and mucositis in the prepandemic cohort were detected at baseline. These are risk factors for HAIs, particularly for CLAMBIs, and this could have contributed to a trend toward higher rates of CLAMBIs in that cohort. However, 3 polymicrobial CLAMBIs occurred in the prepandemic cohort and none during the pandemic. A polymicrobial central-line infection can occur from contamination, so it is logical that these would decrease with improved hygiene practices.

Importantly, we detected a significant decrease in the proportion of patients with a positive respiratory virus test. The additional infection prevention practices were put in place to decrease transmission of respiratory viruses, such as SARS-CoV-2. These results are encouraging with regard to the effectiveness of these measures in preventing the spread of respiratory viruses, especially in the community setting because most respiratory virus tests were obtained on admission. Notably, this finding was not due to more or fewer respiratory virus tests being run during the pandemic. During the study period, all SARS-CoV-2 tests at our institution were run independently from the respiratory virus panels, which test for viruses such as adenovirus and rhinovirus, among others. The number of respiratory viral tests sent, whether positive or negative, was comparable between the 2 cohorts.

This study had several limitations. It was a single-center, retrospective study with a small sample size, so the results may not be generalizable to other centers. We attempted to account for the small sample size by reporting infections per 1,000 inpatient hospital days. However, we may not have detected a difference due to the overall low rates of infections in both cohorts. Additionally, as noted previously, differences in baseline characteristics, such as mucositis and GVHD, could have placed the prepandemic cohort at a higher risk for infections at baseline.

In conclusion, in our study, there was no significant difference in HAI rates for malignant hematology and stem cell transplant patients during the COVID-19 pandemic. However, we did note lower rates of positive respiratory virus panels on admission in the context of widespread infection control measures in the community. Regarding risks, benefits, and costs, whether to maintain current hospital infection prevention practices in this high-risk population or to return to the pre–COVID-19 standard of care remains a question for further study. Future randomized or prospective studies could analyze (1) the impact of infection prevention practices in strictly neutropenic populations who are at highest risk for HAIs, (2) the impact of individual control measures such as mask use or improved universal hand hygiene, or (3) specialized prevention of individual types of HAIs such as CDIs versus CLABSIs or CLAMBIs.
